# Perspectives of a Nigerian former WHO-HQ intern

**DOI:** 10.11604/pamj.2015.22.42.7140

**Published:** 2015-09-17

**Authors:** Oluwaseyi Tobi Owaseye

**Affiliations:** 1 University of Ibadan, Nigeria; 2 Network of WHO Intern Alumni

**Keywords:** Internship, experience, access

## Opinion

My name is Oluwaseyi Owaseye. I am a Nigerian. I graduated with a B.Sc. degree in Child Development and family Studies from the Federal University of Agriculture, Abeokuta in 2009. Thereafter, I proceeded to the University of Ibadan, where I studied Public Health (MPH).

As a requirement for my MPH degree and my desire to gain experience in global health, I applied for the WHO Internship programme in the summer of 2012 and I was offered an Internship placement into the Prevention of Blindness and Deafness Unit of WHO-HQ for a period of three months (10 November 2012 - 9 February 2013). I was determined to seize this once in a lifetime opportunity. However, after several unsuccessful attempts to raise funding for my travel and stay in Geneva for the three month internship, I had to turn down my internship offer. Instead, I requested a winter internship in the hope of having more time to fundraise. I received another offer from the same department to start an internship from February to May 2013. Determined to help me succeed on the second attempt, my parents raised enough money to cover my return flight to Geneva. To raise funds for my stay in Geneva I sold household equipment (furniture and electronic). Yet despite these efforts, the amount was only enough to sustain me for one month in one of Europe's most expensive cities.

I then turned to my church in Nigeria, who kindly wrote on my behalf to the church in Geneva with the hope they would assist me with accommodation. Fortuitously, the pastor of my church in Geneva agreed to house me throughout my Internship period and helped me with food. With the support from family and my church I was able to accept the new offer and successfully undertake a three month Internship. There I helped organize an informal consultation on hearing aid technology in low- and middle- income countries, and supported the production of the 2013 edition of fact sheet on Ear and Hearing Care. Moreover, my department renewed my internship for a further three months.

As a young public health professional, the Internship experience at WHO-HQ was an absolute privilege. It was rewarding for me both personally and professionally, but most importantly it has benefited the health system in my country. After my internship, i applied the global health management experience i'd learned in Geneva as the State Manager of the Ebola Emergency Operation Centre, Lagos, Nigeria.

My story is not unique. There is a rich and diverse pool of talent both across Africa and other developing or emerging countries. But for many young people passionate about global health in these countries, the transformational international experiences I was able to gain – with luck and at much sacrifice – are not accessible. Due to the unpaid nature of WHO-HQ Internships and the high cost of living in Geneva, many qualified students and young professionals are unable to participate in WHO internships, even if they apply and are accepted to the program [1, 2]. The global health community and the United Nations (including WHO) recognise that 80% of the world's population live in developing countries, along with 80% of the global burden of disease. We need access to global health training opportunities such as internships if we are to strengthen our health care systems here at home.

Donate [3] now to a project led by myself and other former WHO-HQ interns from over 15 countries, to support two accepted Low- and Middle- Income Country interns at the World Health Organization Headquarters (WHO-HQ) in Geneva. We'll create a short video documentary and written report of the issues surrounding global health internship access narrated through the supported interns’ experience. Thank you.
